# Impact of Food and Drink Administration Vehicles on Paediatric Formulation Performance Part 2: Dissolution of Montelukast Sodium and Mesalazine Formulations

**DOI:** 10.1208/s12249-020-01815-9

**Published:** 2020-10-15

**Authors:** J. Martir, T. Flanagan, J. Mann, Nikoletta Fotaki

**Affiliations:** 1 Department of Pharmacy and Pharmacology, University of Bath, Claverton Down, Bath, BA2 7AY UK; 2 Oral Product Development, Pharmaceutical Technology & Development, Operations, AstraZeneca, Macclesfield, UK; 3 UCB Pharma, Chemin du Foriest, B - 1420 Braine-l’Alleud, Belgium

**Keywords:** drug manipulation, food, drinks, dissolution, mini-paddle, multivariate analysis, paediatrics

## Abstract

Paediatric medicines are not always age-appropriate, causing problems with dosing, acceptability and adherence. The use of food and drinks as vehicles for medicine co-administration is common practice, yet the impact on drug bioavailability, safety and efficacy remains unaddressed. The aim of this study was to use *in vitro* dissolution testing, under infant simulating conditions, to evaluate the effect of co-administration with vehicles on the dissolution performance of two poorly soluble paediatric drugs. Dissolution studies of mesalazine and montelukast formulations were conducted with mini-paddle apparatus on a two-stage approach: simulated gastric fluid followed by addition of simulated intestinal fluid. The testing scenarios were designed to reflect daily administration practices: direct administration of formulation; formulation co-administered with food and drinks, both immediately after mixing and 4 h after mixing. Drug dissolution was significantly affected by medicine co-administration with vehicles, compared to the direct administration of formulation. Furthermore, differences were observed on drug dissolution when the formulations were mixed with different vehicles of the same subtype. The time between preparation and testing of the drug-vehicle mixture also impacted dissolution behaviour. Drug dissolution was shown to be significantly affected by the physicochemical properties and composition of the vehicles, drug solubility in each vehicle and drug/formulation characteristics. Ultimately, in this study, we show the potential of age-appropriate *in vitro* dissolution testing as a useful biopharmaceutical tool for estimating drug dissolution in conditions relevant to the paediatric population. The setup developed has potential to evaluate the impact of medicine co-administration with vehicles on paediatric formulation performance.

## INTRODUCTION

Paediatric oral drug development and administration remain challenging due to specific age-related problems. Availability of authorised, age-appropriate medicines is limited and there is no general rule of how to safely administer oral medicines to the paediatric population (1,2). Therefore, pharmacists and carers often manipulate adult dosage forms prior to administration.

The use of food and drinks as vehicles for medicine co-administration is common practice to deliver a specific dose and improve compliance, yet the scientific rationale for selecting a particular type of vehicle for mixing with the medicine is often not evident (3–5). The majority of vehicles suggested for medicine co-administration seem to be recommended more on the basis of their taste and texture for the paediatric population rather than their impact on *in vivo* drug product performance. In addition to the possible negative effects on dose accuracy (as often reported (5–7)), drug manipulation and mixing with different food and drinks can also affect drug stability, solubility and bioavailability, ultimately leading to either sub-therapeutic or toxic drug levels (8–10). These effects are still often unaddressed.

It has been shown that different food and drinks can have an effect on paediatric medicine performance. In a recently published study (the first part of this study) conducted by the research group, the physicochemical properties of a selection of (soft) food and drink vehicles, commonly reported to be mixed with paediatric medicines prior to administration, were measured and the impact of the co-administered vehicles on the solubility of two poorly soluble drugs commonly administered to children (montelukast (sodium) and mesalazine) was assessed (11). The solubility of both montelukast and mesalazine was significantly affected by the physicochemical properties (pH, buffer capacity, surface tension, osmolality, viscosity) and macronutrient composition (fat, sugar and protein content) of commonly used vehicles (11). Similarly, medicine co-administration with different vehicles may affect drug dissolution properties to a different extent. Dissolution of amlodipine (BCS class I; weak base, pKa 8.6; logP 3.0 (12)) from crushed tablets mixed with jam has been shown to be slower in comparison with mixing with other vehicles (yoghurt, honey, orange juice and water) (13). Dissolution studies of crushed warfarin (BCS class I; weak acid, pKa 5.1; logP 2.7 (14,15)) and carbamazepine (BCS class II; neutral compound; logP 2.5 (15,16)) tablets mixed with water or orange juice resulted in a faster drug dissolution in comparison with the direct introduction of whole tablets. In comparison, no differences were observed between drug dissolution from crushed tablets mixed with honey, jam or yoghurt and the direct introduction of tablets scenario (13). Compatibility studies of tegaserod (BCS class II; weak base, pKa 9.8; logP 2.6 (15,17)), from crushed tablets mixed with food/drinks (water, apple juice, orange juice and applesauce), revealed that whilst the drug was compatible with the vehicles, the dissolution profiles of the crushed tablets mixed with orange juice and applesauce were not comparable with those of intact tablets (18). The time between preparation and administration of the mixture may also have an effect on drug solubility, drug stability and consequently oral drug absorption (10). This vehicle-impact might be critical for certain medications (*e.g.* when immediate release is needed for a fast-therapeutic action), since food-drug interactions can have a significant impact on drug bioavailability and, consequently, therapeutic efficacy (19,20).

Recently, the FDA issued a draft guidance addressing the recommended approaches for determination of the suitability of the vehicles intended for co-administration of paediatric medicines (21). Guidance is given on vehicle selection, description of standardised *in vitro* methods for evaluating vehicle compatibility and suggestions on product labelling for communication of acceptability of vehicles (21). It is necessary to conduct these investigations in order to fully understand the impact of this practice on drug formulation behaviour and better guide healthcare practitioners, patients and carers regarding medicine co-administration with vehicles, in the paediatric population.

*In vitro* dissolution testing is widely used as a predictive biopharmaceutics tool for drug product performance characterisation. Dissolution tests are used for several applications including the following: assessment of batch-to-batch quality process control and quality assurance, formulation development, identification of food effects on the dissolution and bioavailability of orally administered drugs, and of drug solubility limitations and stability issues (22,23). Dissolution tests have been shown to predict *in vivo* drug behaviour in adults by addressing both medicine administration practices and the physiological gastrointestinal (GI) conditions that can affect drug dissolution (22,24). However, these tests require modifications to assess drug performance in paediatrics. The use of dissolution tests to study the impact of medicine co-administration with vehicles on paediatric drug performance would require the incorporation of age-specific gastrointestinal (GI) tract parameters (namely, pH, media volumes and composition and different dosing scenarios) (25).

**Fig. 1:**
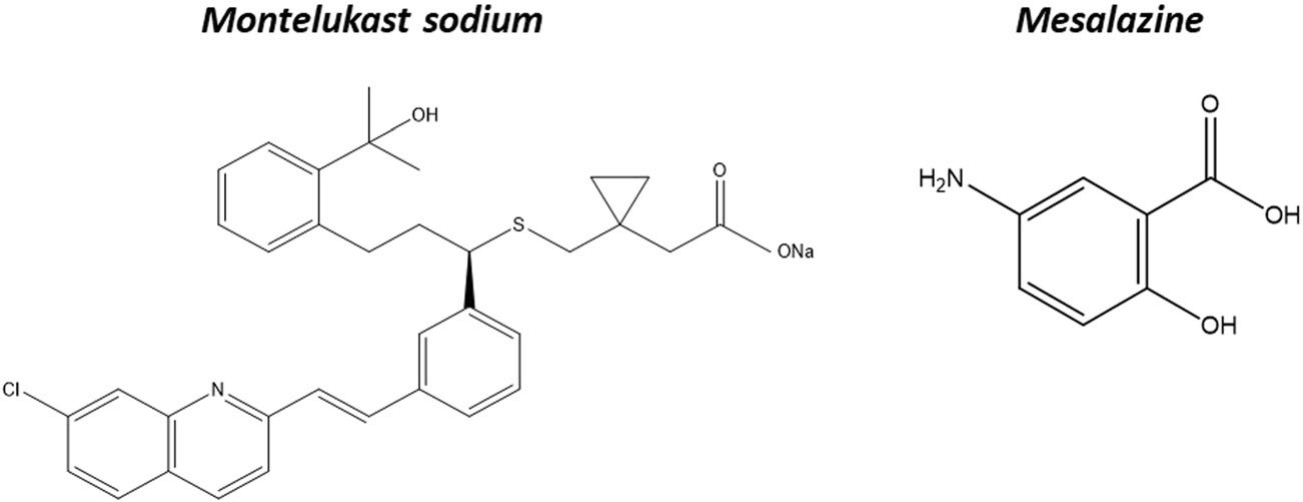
Chemical structure of **a** montelukast sodium and **b** mesalazine (ChemDraw Professional 18.1)

The aims of this study were two-fold: (i) to evaluate the effect of the co-administration with vehicles on the dissolution performance of formulations of two poorly soluble compounds; and (ii) to evaluate the effect of different administration practices (*i.e.* time between preparation and administration of the mixture formulation-vehicle) on drug dissolution. Considering that the fluid volumes available in the GI tract of younger age groups are smaller than in adults, in the present study, an adaptation of the standard USP II apparatus to a mini-paddle apparatus was tested as an appropriate method to address the need for small volume testing. Additionally, a two-stage dissolution protocol was used to simulate the fluid profile (*e.g.* pH, fluid volumes and transit times) when conditions are changed from the gastric to intestinal environment. To our knowledge, little attention has been devoted to the use of such *in vitro* dissolution testing setup to evaluate drug dissolution, whilst addressing typical paediatric dosing conditions such as the effect of medicine co-administration with vehicles. Similar to our first study, montelukast and mesalazine were selected as model compounds; they are poorly soluble compounds, with pH-dependent solubility and recommended to be mixed with vehicles to facilitate paediatric administration. The structures of montelukast sodium and mesalazine are presented in Fig. 1, respectively. Two formulations of each drug were studied (montelukast: Singulair^®^ granules and Actavis^®^ chewable tablets; mesalazine: Pentasa^®^ and Salofalk^®^ granules).

## MATERIALS AND METHODS

### Materials

Ammonium acetate (high-performance liquid chromatography (HPLC) grade), 37% hydrochloric acid, sodium hydroxide, sodium chloride, glacial acetic acid, potassium dihydrogen phosphate, acetonitrile (HPLC grade) and methanol (HPLC grade) were purchased from Fisher Scientific (UK). Trifluoroacetic acid (TFA) (HPLC grade), montelukast sodium (pharmaceutical secondary standard) and mesalazine (≥ 99%) were obtained from Sigma-Aldrich Company Ltd. (UK). Water was ultra-pure (Milli-Q) laboratory grade. Regenerated cellulose (RC) membrane filters (0.45 μm) (Cronus®, UK), and filter papers (0.45 μm), polytetrafluoroethylene (PTFE) filters (0.45 μm) and glass microfiber (GF/D) filters (2.7 μm) (Whatman^®^, UK) were used. Porous full flow polyethylene cannula filters (10 μm) were obtained from Quality Lab Accessories LCC (USA). Nine different soft foods and drinks were used as co-administration vehicles. These were chosen based on differences in their composition, physicochemical properties and drug solubility in each vehicle; these factors are extensively described in our previous study (11). Orange squash, milk U.H.T. full fat and orange juice were purchased from The Co-Operative (UK). Blackcurrant squash was from Lucozade Ribena Suntory Ltd. (UK). First Infant Milk (cow’s milk-based formula) was from Cow & Gate (UK). Applesauces were Bramley applesauce Colman’s of Norwich (referred to as ‘applesauce UK’) from Unilever (UK) and Apfelmark applesauce (referred to as ‘applesauce DE’) from Bauck Hof (Germany). Plain yoghurt from Yeo Valley (UK) and Greek yoghurt from Fage (Greece) were also used. The four formulations studied were kindly donated by AstraZeneca (UK). Product information is summarised in Table I.

**Table I:** Information of the Formulations Used in This Study

Active principal ingredient (API)	Brand	Manufacturer	Formulation type/release mechanism	Excipients	Dose tested (mg)	Administration recommendations (26)
Mesalazine	Salofalk^®^	Dr. Falk Pharma (UK)	Granules/delayed release pH-dependent	Aspartame (E 951), carmellose sodium, cellulose, citric acid, silica, hypromellose, magnesium stearate, Eudragit L 100, Eudragit NE 40 D containing 2% nonoxynol 100, povidone K 25, simeticone, sorbic acid, talc, titanium dioxide (E 171), triethyl citrate, vanilla custard flavour	135	Granules should be placed on the tongue and washed down with water without chewing.
	Pentasa^®^	Ferring (UK)	Granules/extended release pH-independent coating	Ethylcellulose, povidone	135	Granules should be placed on the tongue and washed down with water or orange juice, without chewing. Contents of one sachet should be weighed and divided immediately before use; any remaining granules should be discarded.
Montelukast	Singulair^®^	Merck Sharp & Dohme Ltd. (UK)	Granules/immediate release	Mannitol, hydroxy-propyl cellulose, magnesium stearate	4	Granules may be swallowed or mixed with cold, soft foods (not liquid), and taken immediately.
	Actavis^®^	Actavis (UK)	Chewable tablets/immediate release	Lactose monohydrate, aspartame	5	Tablet should not be taken with food; should be taken at least 1 h before or 2 h after food.

### Methods

#### Dissolution Media Preparation

Simulated gastric fluid *sine pepsin* (SGF*sp*) pH 1.2 and simulated intestinal fluid (SIF*sp*) were prepared according to the USP recipes (27). Double concentrated SIF*sp* containing an additional amount of sodium hydroxide (to neutralise the acid present on the first step) was prepared for the two-stage dissolution studies performed.

#### Sample Preparation

Squashes and formula were prepared as per manufacturer’s instructions (formula: 1 scoop of powder (approximately 4.5 g) was added to 30 mL of boiled cooled water; squashes: 25 (orange) or 50 (blackcurrant) mL of concentrated product were diluted in 250 mL of water).

For the direct administration scenario, the formulations were introduced in the media without being mixed with a vehicle. For the scenario of mixing the formulations with vehicles, each sample was prepared by addition of the formulations (corresponding to the ‘dose tested’ in Table I) to 25 mL of drink or 10 mL (approximately 10 g) of soft food, at room temperature. All samples were then manually mixed with a stainless-steel spatula, for 30 s. Actavis^®^ chewable tablets were crushed prior to being mixed with the vehicles or tested.

To test different administration practices, samples with vehicles were prepared as described above and set aside (at room temperature and protected from direct light), and after 4 h, they were remixed with a stainless-steel spatula, prior to performing the study.

#### *In Vitro* Dissolution Studies

Dissolution studies were performed with a mini-paddle apparatus (Agilent Technologies 708-DS apparatus configured with TruAlign 200-mL vessels and electropolished stainless steel mini-paddles; Agilent, USA). Experiments were conducted in a two-stage approach: in SGF*sp* pH 1.2 (total volume with sample: 100 mL), for 1 h, followed by SIF*sp* pH 6.8 (final volume: 200 mL), for 3 h. A dissolution study with a sequential media change mimics the passage of oral dosage forms through the GI tract, providing an understanding of the *in vivo* drug performance (28). Experiments were conducted at 37 ± 0.5°C and the agitation rate of the mini-paddle was set to 50 revolutions per minute (rpm). Sample collection took place at 5, 15, 30, 45, 60, 75, 90, 120, 180 and 240 min. Two-millilitre samples were withdrawn (with volume replacement with the corresponding media), using a 2-mL glass syringe (Fortuna Optima^®^ fitted with a stainless tubing) through a cannula fitted with a full flow filter (10 μm). All experiments were performed without direct light exposure to avoid photodegradation of the drugs (29,30). After collection, samples were filtered through a GF/D (2.7 μm) filter and treated. Sample treatment was as follows: 1000 μL of acetonitrile (montelukast) or 10% (v/v) TFA/water (mesalazine) were added to 500 μL of sample. This mixture was vortexed (HTZ, UK) for 1 min and centrifuged (8000 rpm, 15 min, 4°C) (Beckman Coulter J2-MC centrifuge, UK). The supernatant was filtered through a RC (montelukast) or PTFE (mesalazine) (0.45 μm) filter, placed in an HPLC amber vial and analysed. The pH of the media was measured at the end of each experiment.

The effect of different administration scenarios and testing conditions was investigated by varying the dissolution test parameters, as described in Table II. These were as follows: (1) effect of co-administration of formulation with selected vehicles in comparison to direct administration of formulation; (2) effect of different mixing patterns (*i.e.* time between preparation and administration/testing of the formulation-vehicle mixture); and (3) effect of hydrodynamics (50 *vs* 100 rpm, in selected studies of Pentasa^®^ and Singulair^®^ granules).

**Table II:** Dosing Scenarios and Testing Conditions Investigated

Setup	Agitation speed (rpm)	Scenario: direct introduction	Scenario: mixing with vehicles	Formulations	Mixing pattern (h)
1	50	✓	M, OJ, BLS, PY, APS_UK,_ F, OS, GY, APS_DE_	All	0
2	50	N/A	M, OJ, BLS, PY, APS_UK_	All	4
3	100	✓	M, OJ, BLS, PY, APS_UK_	Singulair^®^, Pentasa^®^	0

*BLS*, blackcurrant squash; *OS*, orange squash; *M*, milk; *F*, formula; *OJ*, orange juice; *PY*, plain yoghurt; *GY*, Greek yoghurt; *APS*_*UK*_, applesauce UK; *APS*_*DE*_, applesauce DE; *N/A*, not applicable

All experiments were performed in triplicate. Fresh calibration curves (concentration range: 0.5–100 μg/mL (montelukast) and 0.5–200 μg/mL (mesalazine)) were prepared in the corresponding media, by appropriate dilution of a 1000 μg/mL stock solution of the analytical standard in methanol (montelukast) or 0.05% TFA/water (mesalazine); the same treatment process was applied as described for the samples. Results were expressed as mean percentage of drug dissolved ± standard deviation (S.D.), at the given sampling time.

#### Chromatographic Conditions for Drug Analysis

The chromatographic methods used for drug analysis were modifications of published methods (31,32). Drug quantification was performed with HPLC with ultraviolet (UV) detection (Agilent HPLC system 1100/1200 series; Agilent, USA). A RP Agilent Eclipse XDB C_18_ column (250 mm × 4.6 mm, 5-μm particle size) was used for both drugs. For montelukast, the mobile phase was composed of ammonium acetate buffer pH 5.5 and methanol (solvents A and B, respectively) delivered at a flow rate of 1 mL min^−1^, on a linear gradient. The selected gradient started with 10% of solvent B, which was increased to 50% within 2 min, and 90% within 4 min; at 11.30 min, the initial conditions of analysis were re-established. Injection volume was 100 μL. Analysis was performed at 20°C and the detection wavelength was 284 nm. Elution time for montelukast was 8.9 min. For analysis of mesalazine, the mobile phase was composed of 0.05% TFA/water and methanol (95:5), delivered at a flow rate of 1 mL min^−1^. Injection volume was 20 μL. Analysis was performed at 40°C and the detection wavelength was 304 nm. Elution time for mesalazine was 4.6 min.

#### Statistical Analysis of Dissolution Data

To describe and compare the dissolution profiles obtained, linear trapezoidal method was used to calculate the area under the curve of each profile over 4 h (AUC_0–4h_). This allowed the use of one value representative of drug dissolution to compare the different scenarios tested.

One-way analysis of variance (ANOVA) with a post hoc Tukey honest significant difference (HSD) test was conducted to investigate statistically significant differences (*p* < 0.05 noting significance level) in the AUC_0–4h_ between direct administration of formulation and mixing the formulations with the different vehicles. *t* test analysis was used to compare AUC_0–4h_ results obtained between drug dissolution after mixing the formulations with vehicles of same subtype or drug dissolution after mixing the formulations with the same vehicle under different testing conditions (*i.e.* agitation rate or time between preparation and mixing) (*p* < 0.05 noting significance). The analyses were performed with GraphPad Prism® v.7 software (San Diego, USA).

Partial least squares regression (PLS-R) analysis was used to correlate the AUC_0–4h_ values of the different testing scenarios (response factor) with the physicochemical properties and macronutrient composition of the vehicles (pH, buffer capacity, surface tension, viscosity, osmolality; percentage of fat, sugars and proteins), drug solubility in each vehicle, type of formulation and testing conditions (*i.e.* preparation time) (XLSTAT Software; an Add-In for Excel, Microsoft^®^). The physicochemical properties and macronutrient composition of the vehicles and well as drug solubility values in each vehicle were previously presented (11). When analysing both drugs together, drug characteristics (logP (log octanol-water partition coefficient) and ionisation percentage (obtained from ACD/Labs© 2010–2018)) were also considered as variables. The quality of the model was evaluated with the square of the coefficient of determination (*R*^2^) and goodness of prediction (*Q*^2^), with values close to 1 being indicative of good fit and prediction power, respectively (33). Full cross-validation (leave-one-out procedure) was used to develop and evaluate the regression model. The optimum number of calibration factors for each model was selected based on the optimum predictability of the model and predicted residual error sum of squares (PRESS). The standardised coefficients of the factors indicated the relative effect (positive or negative) of their corresponding variables on the response. The variable importance in projection (VIP) value was used to evaluate the importance of each factor on the model (33). Model variables with VIP values > 1 were evaluated as the most important in explaining the variation in the dependent variable, whilst values between 0.7 and 1 were considered moderately influential for the model. Values < 0.7 were deemed not of significance for the prediction of the dependent variable (33).

## RESULTS AND DISCUSSION

### Effect of Medicine Co-administration with Food and Drinks on Drug Dissolution

**Fig. 2:**
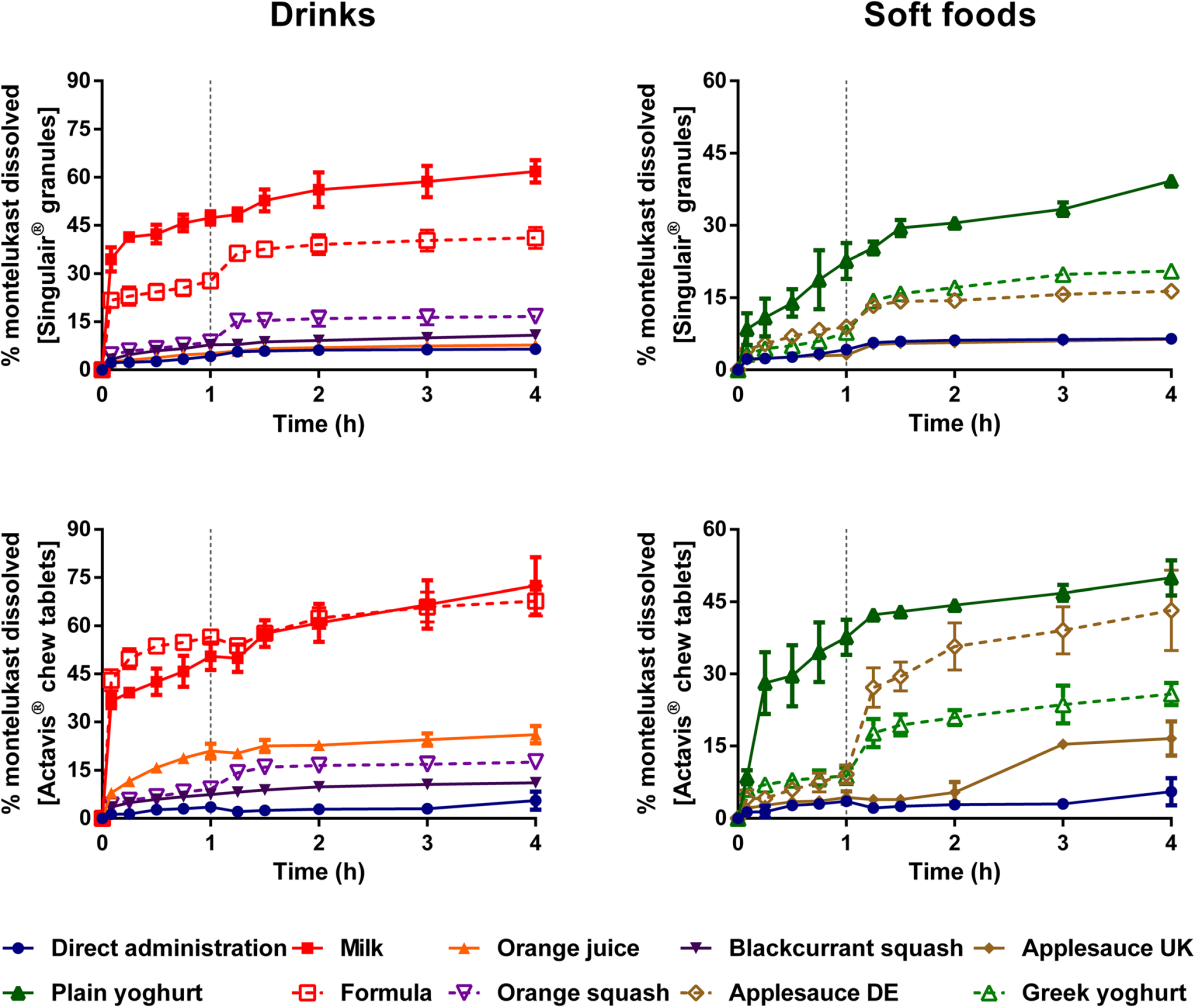
Mean percentage of montelukast dissolved (± S.D.) from Singulair^®^ granules (top panel) and Actavis^®^ chewable tablets (bottom panel) after direct administration of formulation, after mixing with selected vehicles (full lines) and with vehicles of the same subtype (dashed lines). Dotted vertical lines represent the time of medium change

Dissolution of montelukast from the two formulations revealed a significant effect of medicine co-administration with food and drink vehicles, compared with the direct administration scenario (Figs. 2 and 4).

For Singulair^®^ granules, the AUC_0–4h_ was significantly lower for the direct administration scenario compared with when the granules were mixed with vehicles, except for orange juice, blackcurrant squash and applesauce UK. For the co-administration with drinks scenario, drug dissolution was higher at 4 h when the formulation was mixed with milk (61.9%), followed by when it was mixed with formula, orange squash, blackcurrant squash and orange juice (percentage of drug dissolved = 41.1, 16.7, 10.8 and 7.7, respectively). For an ionisable compound like montelukast (amphoteric; pKa _basic_ 2.7 and pKa _acidic_ 5.8 (34)), an increase in pH can affect the ionisation percentage of the drug. Therefore, drug solubilisation and dissolution are higher when the formulation is mixed with a dairy drink (pH between 6.5 and 6.8 (11)) in comparison with other vehicles due to an increase in the drug ionisation percentage. For co-administration with soft foods, the highest drug dissolution was observed when the granules were mixed with plain yoghurt (39.3%) and the lowest when the formulation was mixed with applesauce UK (6.4%). Drug dissolution differed when vehicles of the same subtype were tested (AUC_0–4h_ differed between milk/formula, and between squashes, yoghurts and applesauces; *p* < 0.05). The pH of the dissolution media at 4 h was 6.8 ± 0.15 for all tested scenarios. The lower drug dissolution observed when the granules were mixed with applesauce UK, in comparison with when mixed with the other soft foods, is probably due to the presence of starch in its composition, which forms a net gel around the formulation that is strengthened by fruit pieces and negatively impacts drug dissolution (13). Results show that vehicles of the same type (*e.g.* soft foods) have a distinct impact on drug dissolution (*e.g.* extremely low to no drug dissolution in the case of mixing with applesauce UK but not when formulation was mixed with plain yoghurt) and it can be hypothesised that this vehicle-impact may, ultimately, affect drug behaviour *in vitro*. This is of particular importance considering that the recommendations for administration of Singulair^®^ granules are to mix with ‘a spoonful of cold soft foods’ (26). Therefore, the differences observed in drug dissolution indicate the potential risk of not following vehicle recommendations in clinical practice. Moreover, when evaluating vehicle suitability during drug development, the physicochemical properties of the vehicles should be considered.

Dissolution of the crushed Actavis^®^ chewable tablets mixed with vehicles resulted in a higher percentage of drug dissolved at 4 h and significantly higher AUC_0–4h_ in comparison with direct administration of the crushed formulation. Amongst vehicles, drug dissolution was the highest when the crushed tablets were mixed with milk and formula (72.5 and 67.8%, respectively) and the lowest when the formulation was mixed with blackcurrant squash (11.1%). Significant differences in AUC_0–4h_ were revealed between mixing the formulation with milky and fruity drinks, and between mixing with the different squashes tested. Twofold differences were observed between AUC_0–4h_ when mixing with vehicles of the same subtype (plain in comparison with Greek yoghurt and between the applesauce UK and applesauce USA; *p* < 0.05). These differences could be attributed to the physicochemical properties (*e.g.* different pH and protein content of dairy *vs* fruity drinks, and different viscosity of the applesauces) and macronutrient composition of the vehicles (*e.g.* different sugar content between the squashes), which affect drug solubilisation and may impact drug dissolution behaviour (11).

**Fig. 3:**
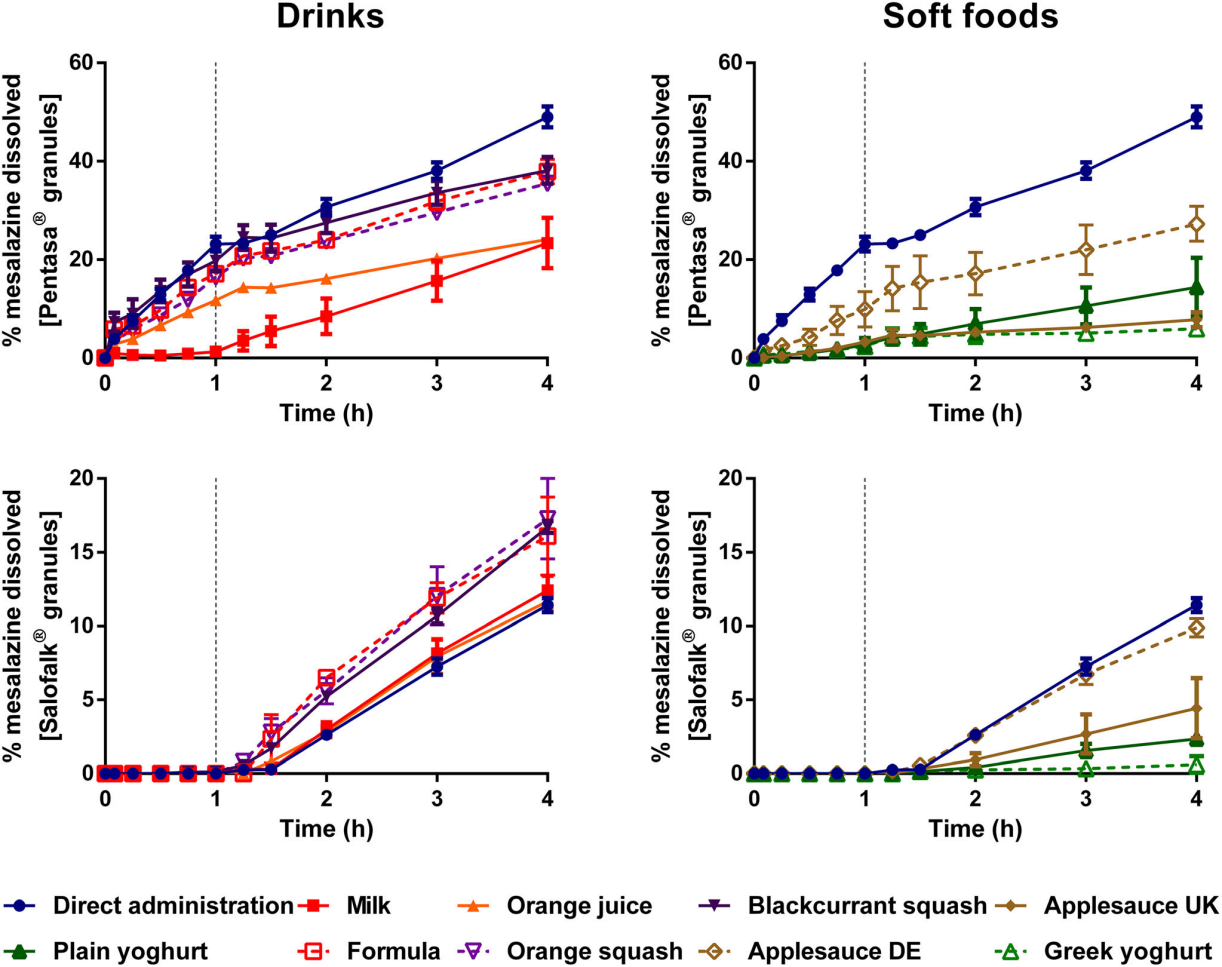
Mean percentage of mesalazine dissolved (± S.D.) from Pentasa^®^ (top panel) and Salofalk^®^ granules (bottom panel) after direct administration of formulation, after mixing with selected vehicles (full lines) and with vehicles of the same subtype (dashed lines). Dotted vertical lines represent the time of medium change

**Fig. 4:**
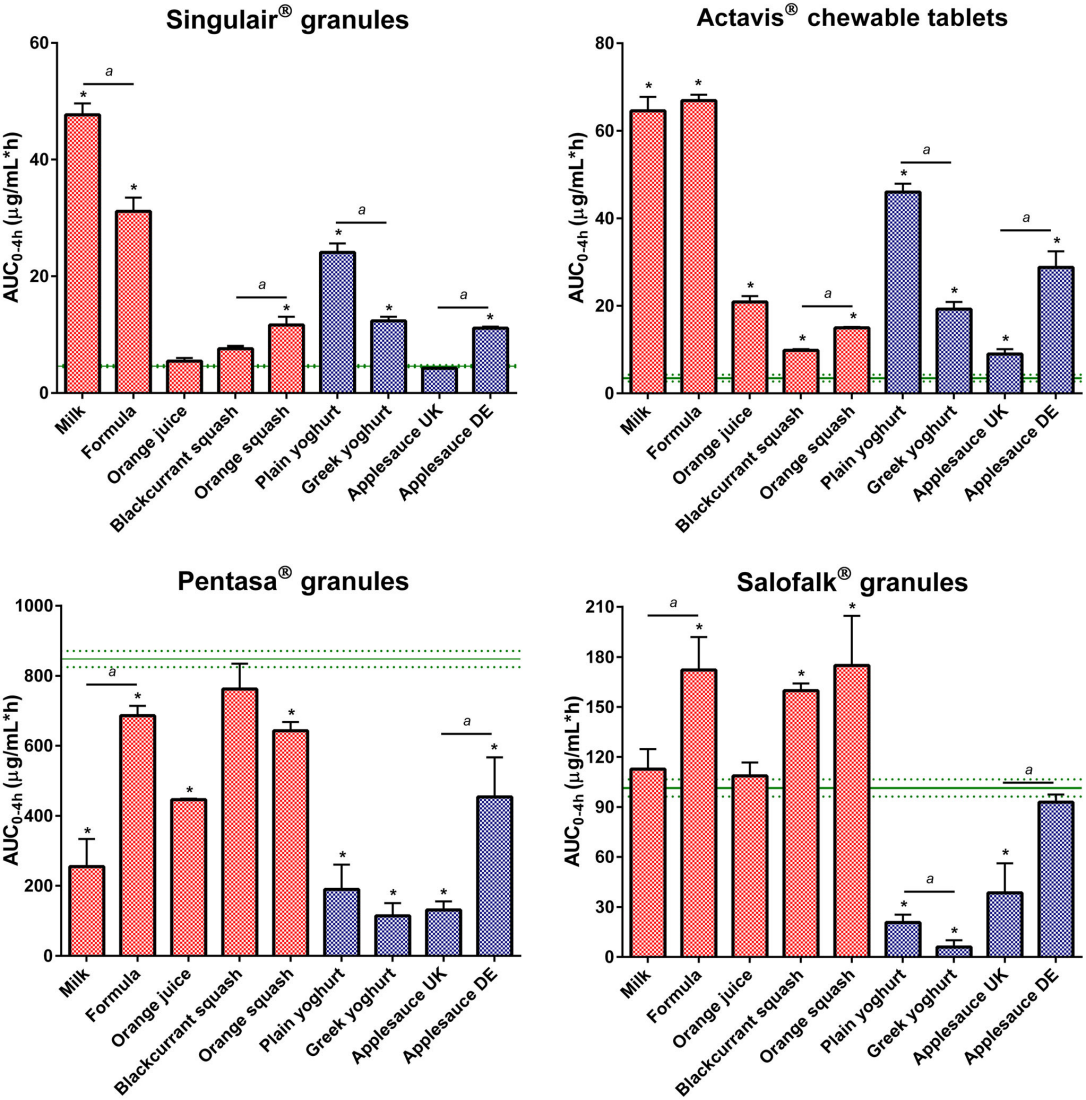
Effect of co-administration of formulation with vehicles on percentage of drug dissolved at 4 h from the tested formulations. Asterisk symbol denotes a statistical difference on drug dissolution between direct administration (dashed line) and co-administration with vehicles (bars; red: drinks, blue: soft foods). *a* denotes statistical difference when vehicles of the same subtype were tested (*p* < 0.05)

Dissolution of mesalazine from Pentasa^®^ and Salofalk^®^ granules also revealed a significant effect of co-administration with food and drink vehicles (Figs. 3 and 4).

For Pentasa^®^ granules, co-administration with the different vehicles resulted in a lower percentage of drug dissolved at 4 h compared with the direct administration scenario. The calculated AUC_0–4h_ were significantly lower when the formulation was mixed with vehicles, except blackcurrant squash (*p* < 0.05). For co-administration with drinks, the percentage of drug dissolved (4 h) was higher for mixing with blackcurrant squash and formula (38.1 and 37.9%, respectively) followed by orange squash (35.5%), orange juice (24.1%) and milk (23.4%). Significant differences in AUC_0–4h_ were revealed between mixing the Pentasa^®^ granules with milk and formula (*p* < 0.05), but not between mixing with the different squashes tested. For co-administration with soft foods, AUC_0–4h_ significantly differed when mixing with the different applesauces demonstrating that vehicles of the same subtype can distinctly affect dissolution of different drugs.

The percentage of mesalazine dissolved from Salofalk^®^ granules (4 h) was also affected by the different vehicles. The percentage of drug dissolution was the highest when the granules were mixed with orange squash (17.3%), followed by blackcurrant squash (16.7%), formula (16.1%), milk (12.4%), orange juice (11.7%), direct introduction (11.4%) and soft foods. AUC_0–4h_ was significantly different for the direct administration scenario compared with co-administration with vehicles, except milk, orange juice and applesauce DE (*p* > 0.05). For co-administration with soft foods, the percentage of drug dissolved at 4 h was the lowest when the formulation was mixed with Greek yoghurt (0.6%) and the highest when mixed with applesauce DE (9.9%), indicating that vehicles of the same type (*e.g.* soft foods) have a distinct impact on drug dissolution. The lower drug dissolution observed when the granules were mixed with soft foods was likely due to a physical barrier that these vehicles create around the formulation, which prevents mixing with GI fluids and hinders drug release, ultimately, reducing drug exposure at the site of absorption (13). Dissolution of mesalazine from Salofalk^®^ granules also differed when mixed with vehicles of the same subtype, namely, applesauce UK *vs* USA, milk *vs* formula and plain *vs* Greek yoghurt (*p* < 0.05).

Interestingly, the two mesalazine formulations were oppositely affected when mixed with drinks compared with direct introduction of the granules: for Pentasa^®^, drug dissolution was lower, whereas for Salofalk^®^ granules was higher. The mode of drug release of the two formulations is different; Pentasa^®^ granules have a pH-independent extended release, whereas Salofalk^®^ granules have a pH-dependent delayed release (Table I). Therefore, the vehicle-impact on drug dissolution from different formulations will depend not only on vehicle properties but also on formulation properties (*e.g.* differences in the mode of drug release, type of dosage form).

Overall, it was possible to observe a significant effect of medicine co-administration with soft foods and drinks on the dissolution of both drugs from all the formulations tested. Results show a vehicle-induced impact on drug dissolution due to changes in drug ionisation percentage and, consequently, drug solubility (*e.g.* higher percentage of montelukast dissolved when formulation is mixed with milk), changes in formulation environment (*e.g.* higher viscosity of applesauce hindering drug release/dissolution) and alteration of formulation factors (*e.g.* different coating of the tested mesalazine granules).

### Assessment of the Impact of Different Administration Practices on Drug Dissolution Behaviour

**Fig. 5:**
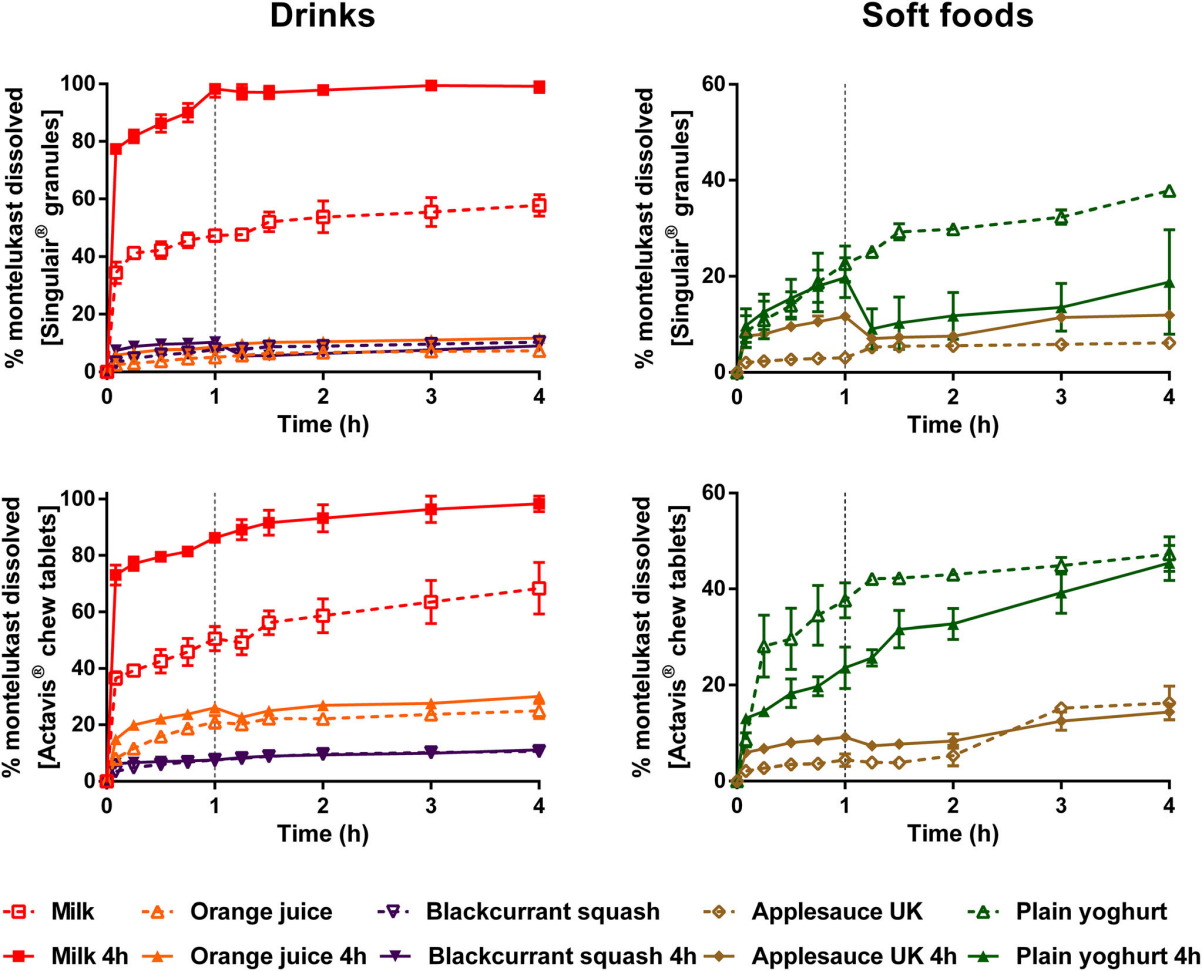
Mean percentage of montelukast dissolved (± S.D.) from Singulair^®^ granules (top panel) and Actavis^®^ chewable tablets (bottom panel), under two administration scenarios: testing immediately after mixing (dashed lines) and 4 h after mixing (full lines). Dotted vertical lines represent the time of medium change

**Fig. 6:**
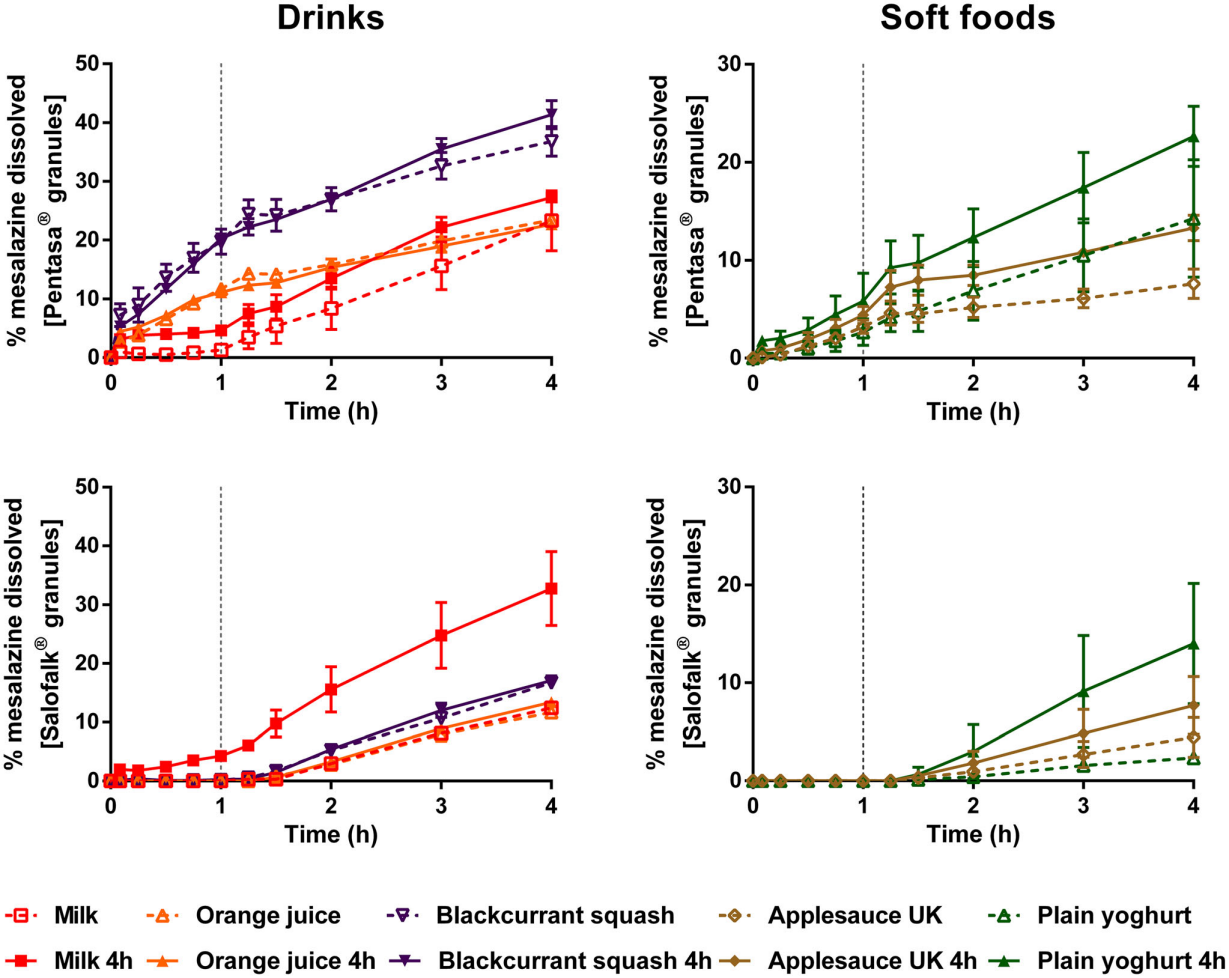
Mean percentage of mesalazine dissolved (± S.D.) from Pentasa^®^ granules (top panel) and Salofalk^®^ granules (bottom panel) under two administration scenarios: testing immediately after mixing (dashed lines) and 4 h after mixing (full lines). Dotted vertical lines represent the time of medium change

**Fig. 7:**
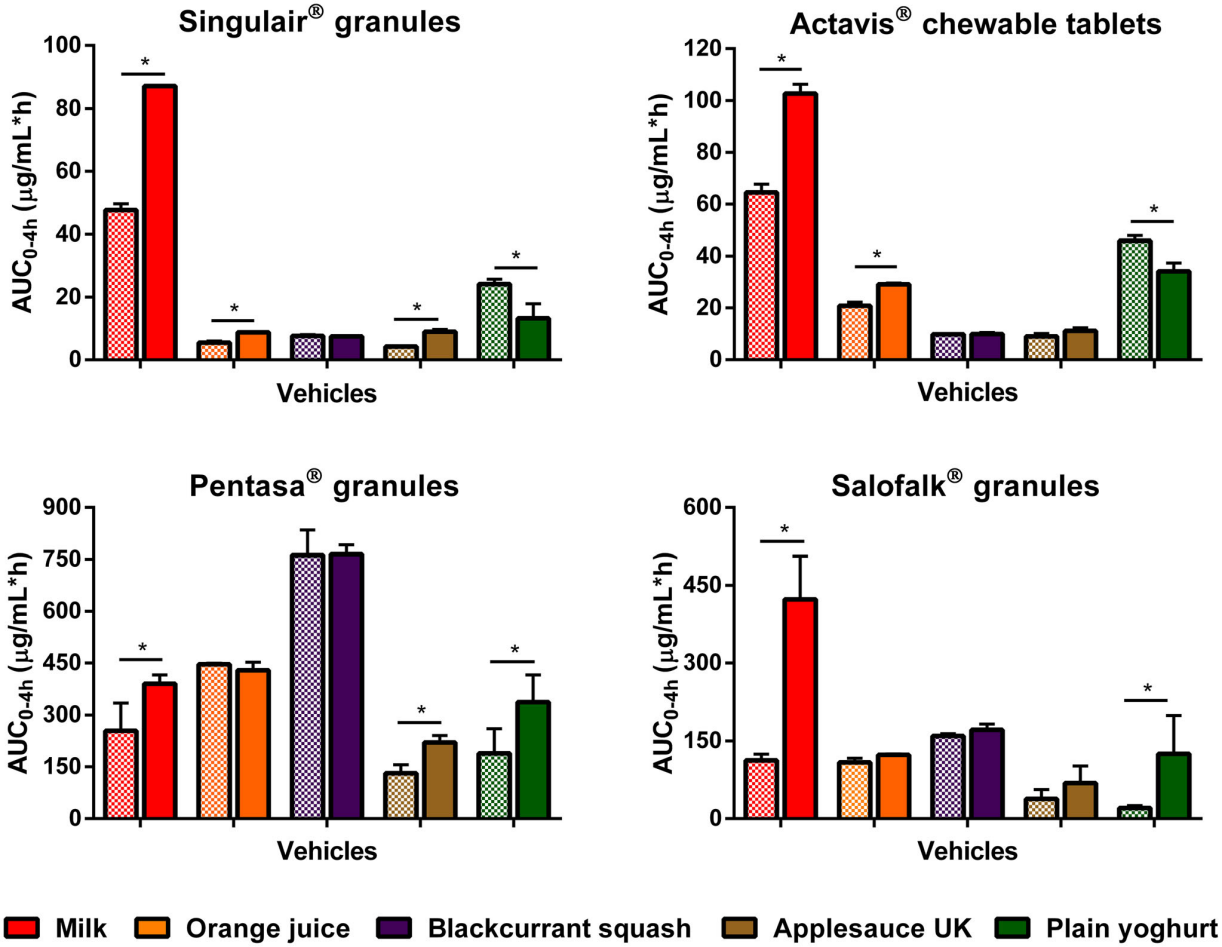
Effect of a 4-h delay between mixing and testing of formulation with vehicles on drug dissolution from the tested formulations. Asterisk symbol denotes a statistical difference on drug dissolution between testing immediately after mixing (dashed bars) and testing 4 h after mixing (full bars)

Delaying testing by 4 h after mixture preparation revealed significant differences on drug dissolution in comparison with testing immediately after mixing (montelukast: Figs. 5 and 7; mesalazine: Figs. 6 and 7).

For Singulair^®^ granules, delaying testing by 4 h after mixing led to a higher percentage of drug dissolved and a significantly higher AUC_0–4h_ for co-administration with milk, orange juice and applesauce UK (*p* < 0.05). This is probably due to the solubility of montelukast in these vehicles, which resulted in an increased drug solubilisation and dissolution during the 4-h delay (11). From the three cases, the differences in drug dissolution between the two testing scenarios were most accentuated when the granules were mixed with milk. As observed in the ‘Effect of Medicine Co-administration with Food and Drinks on Drug Dissolution’ section, this is probably due to the pH of milk which leads to an increase in drug solubilisation and dissolution, in comparison with when the granules are mixed with other vehicles. Results from this test show that this increase is even more evident if there is a delay between preparation and administration of the mixture. In contrast, delaying testing after mixing the Singulair^®^ granules with blackcurrant squash had no effect on drug dissolution, whereas delaying the time between mixing with plain yoghurt and testing resulted in a significantly lower AUC_0–4h_. Delaying testing by 4 h after mixing the granules with applesauce UK and plain yoghurt led to a drop in drug concentration after the media change from SGF*sp* pH 1.2 to SIF*sp* pH 6.8. This might be related to the sudden change in pH and increase in media volume (10).

For the crushed Actavis^®^ chewable tablets, a 4-h delay between mixing the formulation with the vehicles and testing, resulted in a higher percentage of drug dissolved for co-administration with milk and orange juice, but not when the formulation was mixed with blackcurrant squash, applesauce and plain yoghurt. AUC_0–4h_ was significantly different when the crushed chewable tablets were mixed with milk, orange juice (both higher) and plain yoghurt (lower), in comparison with immediate administration of the vehicle-formulation mixtures (*p* < 0.05).

For Pentasa^®^ granules, increasing the time between preparation and testing of the granules-vehicle mixtures resulted in a higher percentage of drug dissolved (4 h), and significantly higher AUC_0–4h_, when the formulation was mixed with milk, applesauce UK and plain yoghurt.

For Salofalk^®^ granules, increasing the time between mixing the formulation with milk and testing resulted in a significantly higher AUC_0–4h_ and 3-fold increase on percentage of drug dissolved (4 h), observed from the beginning of dissolution (pH 1.2). The Salofalk^®^ granules have a pH-dependent modified release coating (due to the presence of the coating polymers Eudragit L and NE 40 D which only disintegrate at pH ≥ 6), and therefore no release is intended during the gastric passage. A 4-h delay between mixing the granules with milk (pH 6.8) and testing resulted in a pH-induced loss of integrity of the coating and, consequently, earlier drug release and dissolution. In contrast, the 4-h delay between mixing the Salofalk^®^ granules with the other vehicles (pH between 2 and 4.5 (11)) and testing did not alter drug dissolution in the first hour of the test (pH 1.2) due to the polymer coating. AUC_0–4h_ was significantly higher when delaying testing after mixing the formulation with plain yoghurt (pH 4.5) (*p* < 0.05). This testing scenario was also associated with large variation in dissolution between replicate tests, probably due to the loss of integrity of the formulation during the mixing which resulted in an unimpaired release.

Overall, results indicate that when medicines are co-administered with vehicles, the mixtures should be administered as soon as possible after preparation (unless specific data is available). Immediate administration reduces to the potential risk of dosing errors, exposure to light, hydrolysis, oxygen and microbiological contamination, but also minimises other vehicle-effects on drug dissolution (*e.g.* increased drug solubilisation, potential stability issues). Depending on the formulation, and particularly for enteric-coated dosage forms (case study, Salofalk^®^), delaying administration of the prepared formulation-vehicle mixture could result in changes in drug dissolution behaviour which might alter drug absorption and, consequently, drug safety and efficacy. Other potential consequences of delaying the administration of the drug-vehicle mixture are an increase of the risk of adverse side effects, depending on the drug category (*e.g.* for nonsteroidal anti-inflammatory drugs, it might lead to irritation of the GI mucosa and, ultimately, ulcers) (13).

### Assessing the Impact of *In Vitro* Hydrodynamics

**Fig. 8:**
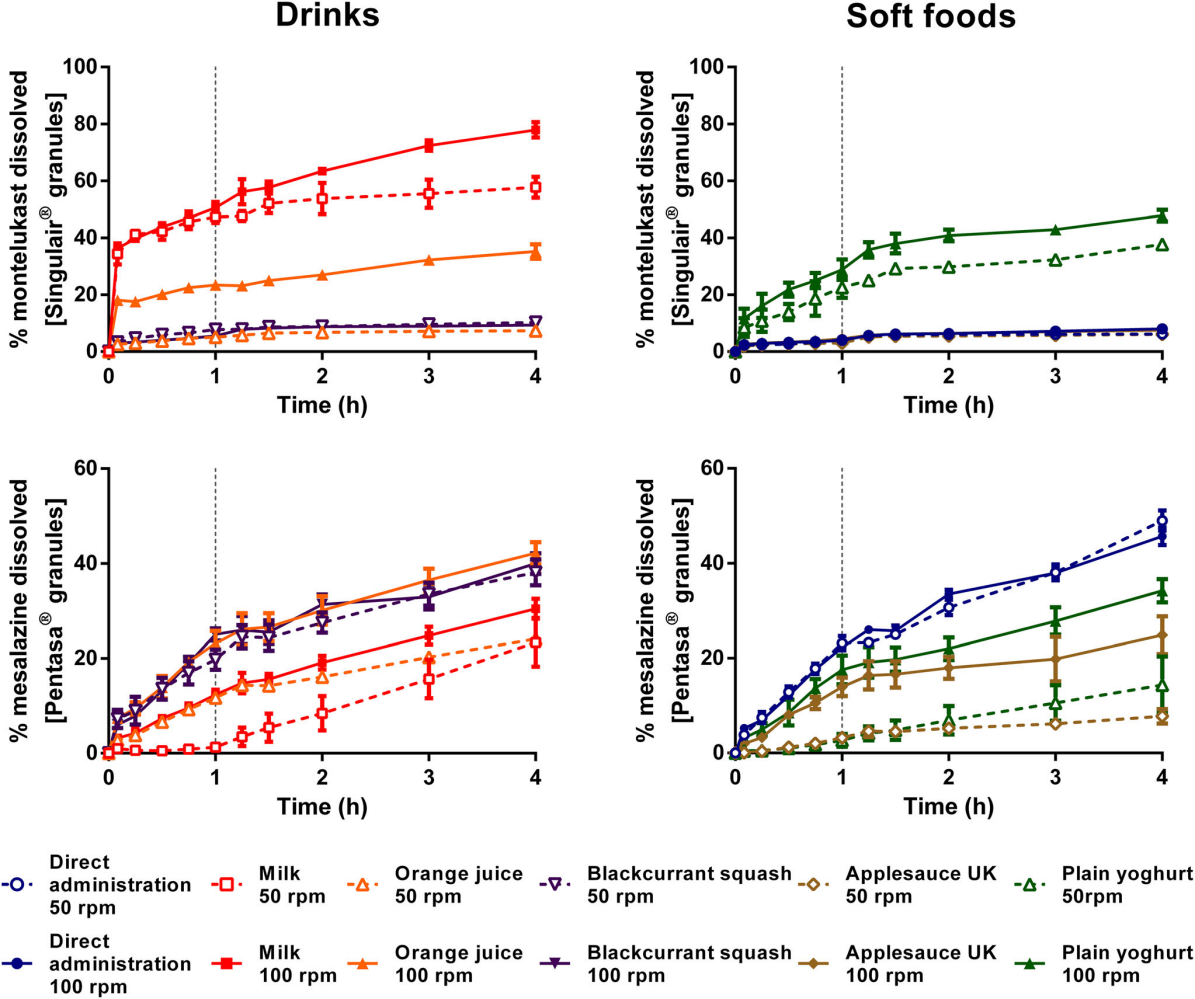
Mean percentage of drug dissolved (± S.D.) of montelukast from Singulair^®^ granules (top panel) and mesalazine from Pentasa^®^ granules (bottom panel), after testing under two agitation rate conditions: 50 rpm (dashed bars) and 100 rpm (full bars). Dotted vertical lines represent the time of medium change

**Fig. 9:**
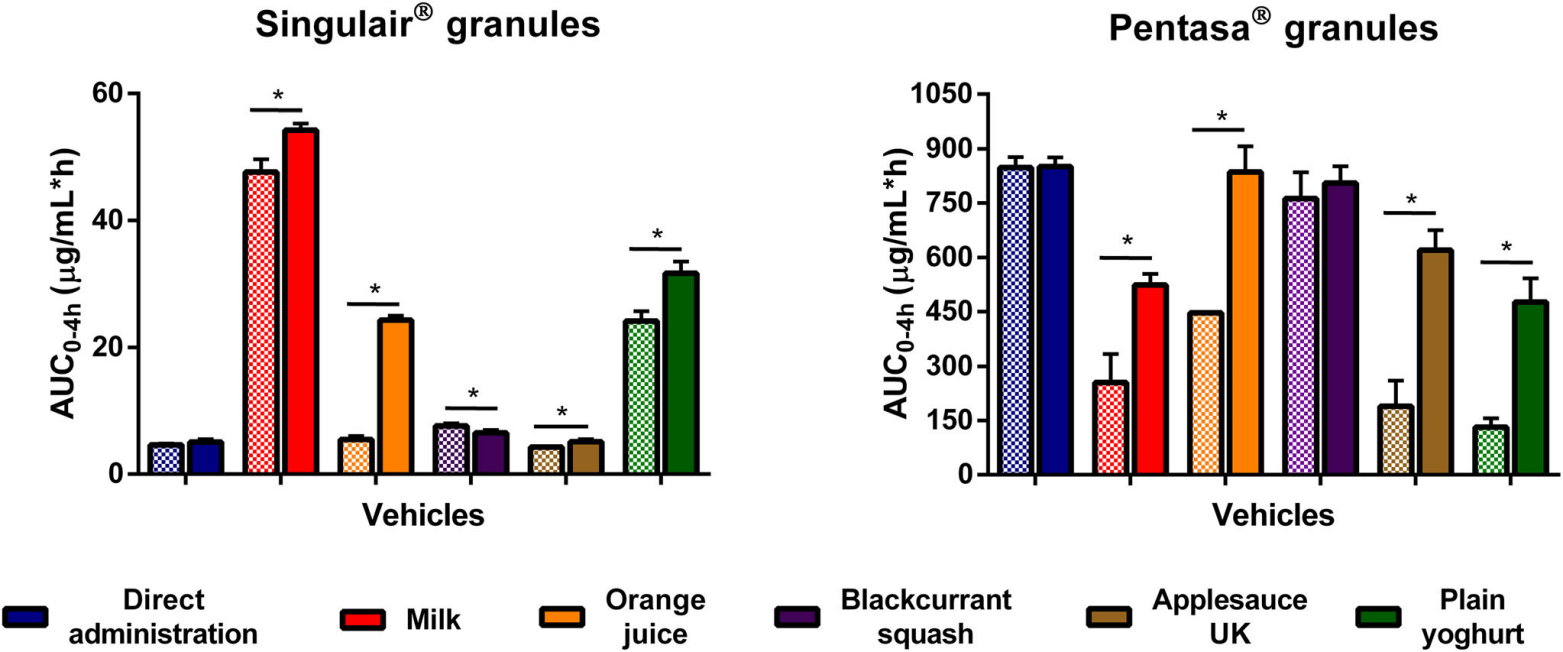
Effect of dissolution hydrodynamics on AUC_0–4 h_ of Singulair^®^ (montelukast) and Pentasa^®^ (mesalazine) granules. Asterisk symbol denotes a statistical difference in AUC_0–4 h_ between drug dissolution when agitation rate was set at 50 (dashed bars) and 100 rpm (full bars)

*In vitro* drug dissolution from the formulations tested (montelukast: Singulair^®^ granules; mesalazine: Pentasa^®^ granules) was influenced by the hydrodynamic conditions, for all the scenarios tested (Figs. 8 and 9).

For Singulair^®^ granules, higher agitation conditions resulted in a higher percentage of drug dissolved (4 h) when the formulation was mixed with milk, orange juice, applesauce and plain yoghurt (increase in percentage of drug dissolved (4 h) = 16.1, 27.5, 1.4 and 8.6%, respectively). A significantly higher AUC_0–4h_ was observed when the granules were mixed with these vehicles and tested at 100 rpm, in comparison with 50 rpm (Fig. 9). This is probably related to the effect of the increased hydrodynamics, which result in a better dispersion of the drug product-vehicle mixture and, consequently, facilitate drug dissolution from the vehicles (35). Dissolution testing of the granules mixed with blackcurrant squash at high agitation rate (100 rpm) resulted in a significantly lower AUC_0–4h_ in comparison with the dissolution testing at low agitation rate (50 rpm.) For the scenario of direct administration, drug dissolution was likely limited by the drug solubility in the media and not significantly affected by the increase in agitation rate (11).

Testing at 100 rpm also resulted in a higher drug dissolution and significantly higher AUC_0–4h,_ when the Pentasa^®^ granules were mixed with milk, orange juice, plain yoghurt and applesauce, compared with 50 rpm (*p* < 0.05).

In comparison with the results obtained when testing at 50 rpm, increasing the agitation rate to 100 rpm resulted in a reduced discrimination between drug dissolution profiles. Nevertheless, comparison of AUC_0–4h_ of the dissolution profiles obtained at 100 rpm still revealed significant differences between direct introduction of the formulation and mixing with vehicles (*p* < 0.05). Therefore, the differences in drug dissolution for co-administration with vehicles in comparison with direct administration of formulation were not due to the agitation speed set (50 rpm) since they were still observed when testing at a higher agitation rate.

The turbulent flow regime generated by USP II apparatus dissolution setups would not represent the relatively nonturbulent *in vivo* conditions (24,36,37). Therefore, differences observed in drug dissolution behaviour *in vitro* might not be observed *in vivo* in cases where peristalsis and contact with the lumen play a role. For example, meal viscosity has resulted in a slower tablet disintegration for BCS class III drugs co-administered with food (38).

### Statistical Evaluation of the Factors Impacting *In Vitro* Drug Dissolution

**Fig. 10:**
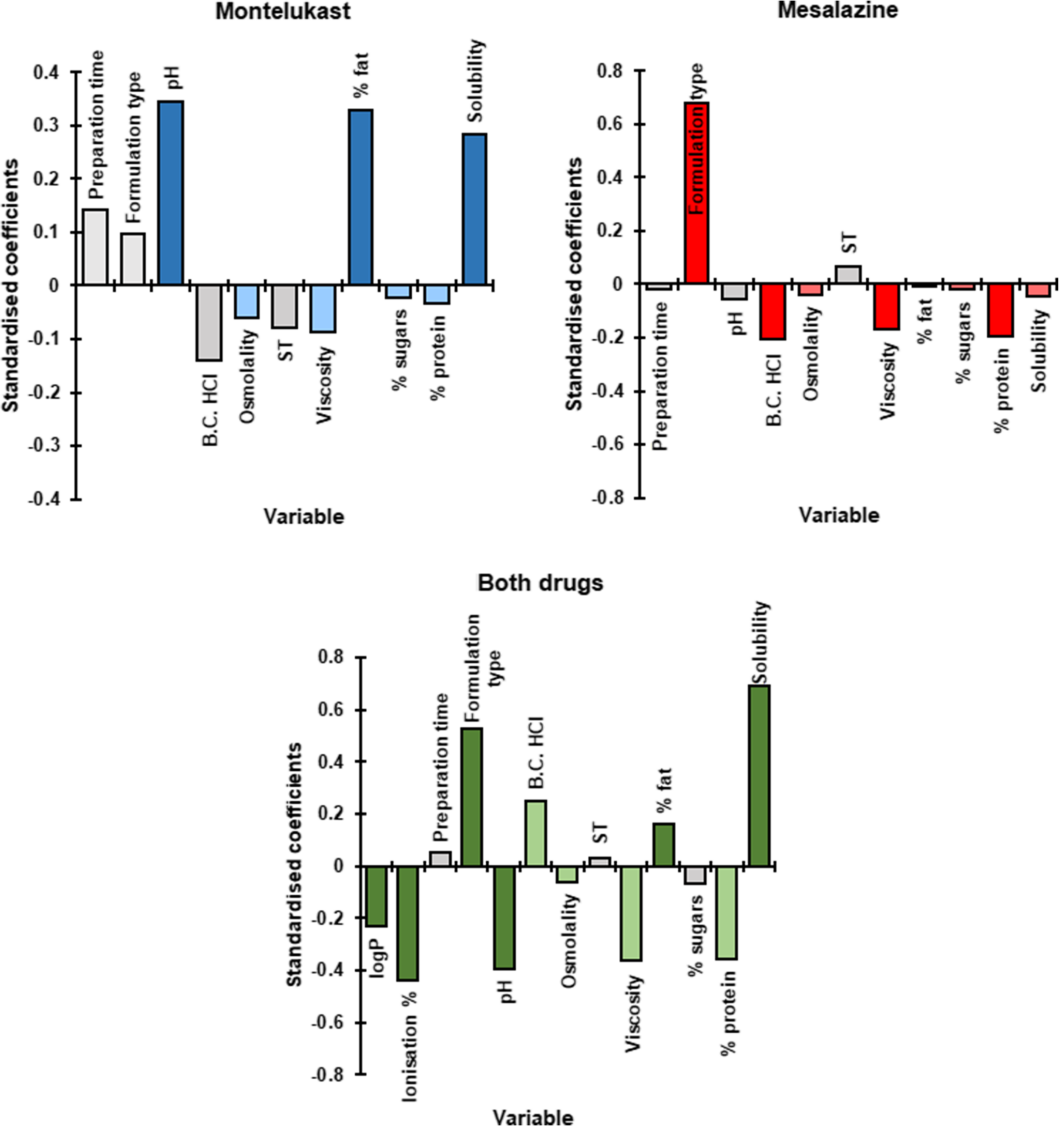
Standardised coefficients corresponding to the variables studied for dissolution of montelukast, mesalazine and both drugs. Colour denotes coefficients with a moderate (lighter colour) and significant (darker colour) impact on the response (VIP > 0.7 and 1, respectively). (B.C., buffer capacity; ST, surface tension)

Results from the PLS-R analyses, conducted to understand the vehicle impact on the dissolution of the two drugs, are shown in Fig. 10.

For the dissolution of montelukast from the granules and crushed chewable tablets, the PLS-R model developed was defined by three components and showed a good fit to the experimental values (*R*^2^ = 0.83) and a good predictive power (*Q*^2^ = 0.79). The statistical analysis revealed that vehicle pH and percentage of fat and drug solubility in each vehicle were the factors with the most significant positive impact on drug dissolution from the two montelukast formulations tested, with a moderate negative impact from vehicle osmolality, viscosity, percentage of sugar and of protein.

For dissolution of mesalazine, the model constructed was defined by two components and showed a good fit to the experimental values (*R*^2^ = 0.70) and a good prediction power (*Q*^2^ = 0.64). PLS-R analysis revealed that the type of formulation was the factor with the most significant positive impact on drug dissolution, whilst significant negative effects from vehicle viscosity, percentage of protein and buffer capacity were observed. Moderate negative effects from vehicle osmolality, percentage of sugars and drug solubility were also observed.

For dissolution of both drugs (all formulations), the PLS-R model built was defined by six compartments, had good predictive power and showed a good fit to the experimental values (*Q*^2^ = 0.62 and *R*^2^ = 0.70, respectively). PLS-R analysis showed that the drug characteristics (logP and ionisation percentage) and the pH of the vehicles were the factors with a significant negative effect on drug dissolution. A moderate negative effect from vehicle buffer capacity was also observed. In contrast, significant positive effects from the type of formulation, drug solubility in each vehicle and percentage of fat of the vehicle were observed. Moderate positive effects from vehicle osmolality, viscosity and percentage of protein were also observed.

Overall, PLS-R results showed that knowledge of the physicochemical properties and macronutrient composition of the food and drinks and drug/formulation properties can help understand the potential vehicle-impact on drug dissolution. This impact should be taken into consideration during compatibility assessments of the vehicle-drug product and could be used to predict potential alterations on drug product behaviour.

## CONCLUSIONS

For poorly soluble drugs, *in vivo* dissolution is likely to be the rate-limiting step of *in vivo* drug absorption and bioavailability. This study aimed to assess the impact of practices of medicine co-administration with food and drinks on the dissolution behaviour of two compounds. Results show that vehicle-induced changes on drug ionisation percentage and solubility (affected by the pH of the different vehicles), formulation environment (*e.g.* higher viscosity of the soft foods) and alteration of formulation factors (*e.g.* different coating of the mesalazine granules) affect drug dissolution behaviour. Drug dissolution was significantly affected by the different vehicles as well as the timing between preparation and testing of the vehicle-drug product mixtures. The use of different vehicles may impact the pharmacokinetic profile of the drug, ultimately altering its clinical performance. For example, alterations in drug bioavailability related to reduced dissolution rates are of concern for drugs that display dissolution as a rate limiting step of absorption, and have a narrow therapeutic index (as the absorbed concentration needed to induce a therapeutic effect may not be reached) or when immediate release is required for fast therapeutic action. Increased drug bioavailability may lead to drug toxicity and adverse clinical side effects. Therefore, it is essential to consider the nature of the vehicles commonly used in practice and the possible effects of different administration recommendations on product performance and, ultimately, clinical performance. The age-appropriate *in vitro* dissolution test used in this study is a useful biopharmaceutical tool for estimating drug dissolution in conditions relevant to infants. Based on the current experimental setup, it is possible to address paediatric administration scenarios (as done in the current study), as well as testing parameters representative of the different paediatric subgroups (*e.g.* by using different volumes, agitation rate and media change times). The dissolution setup described has the potential to provide information on the impact of medicine co-administration with vehicles on paediatric formulation performance and is a useful tool for identifying risks associated with this practice.

## References

[1] Richey RH, Shah UU, Peak M, Craig JV, Ford JL, Barker CE, Nunn AJ, Turner MA (2013). Manipulation of drugs to achieve the required dose is intrinsic to paediatric practice but is not supported by guidelines or evidence. BMC Pediatr.

[2] Turner MA, Catapano M, Hirschfeld S, Giaquinto C (2014). Paediatric drug development: the impact of evolving regulations. Adv Drug Deliv Rev.

[3] Martir J, Flanagan T, Mann J, Fotaki N (2017). Recommended strategies for the oral administration of paediatric medicines with food and drinks in the context of their biopharmaceutical properties: a review. J Pharm Pharmacol.

[4] Akram G, Mullen AB (2012). Paediatric nurses’ knowledge and practice of mixing medication into foodstuff. Int J Pharm Pract.

[5] Venables R, Stirling H, Batchelor H, Marriott J (2015). Problems with oral formulations prescribed to children: a focus group study of healthcare professionals. Int J Clin Pharm.

[6] Ernest TB, Craig J, Nunn A, Salunke S, Tuleu C, Breitkreutz J, Alex R, Hempenstall J (2012). Preparation of medicines for children - a hierarchy of classification. Int J Pharm.

[7] Lippert C, Gbenado S, Qiu C, Lavin B, Kovacs SJ (2005). The bioequivalence of telithromycin administered orally as crushed tablets *versus* tablets swallowed whole. J Clin Pharmacol.

[8] Paradiso LM, Roughead E, Gilbert A, Cosh D, Nation R, Barnes L (2002). Crushing or altering medications: what’s happening in residential aged-care facilities?. Aust J Ageing.

[9] Wells KA, Losin WG (2008). In vitro stability, potency, and dissolution of duloxetine enteric-coated pellets after exposure to applesauce, apple juice, and chocolate pudding. Clin Ther.

[10] Martir J, Flanagan T, Mann J, Fotaki N. Stability of paediatric drugs co-administered with different foods and drinks. 2017. Abstract from AAPS annual meeting, San Diego, USA.

[11] Martir J, Flanagan T, Mann J, Fotaki N (2020). Impact of food and drink administration vehicles on paediatric formulation performance: part 1-effects on solubility of poorly soluble drugs. AAPS PharmSciTech.

[12] Van Zwieten P (1994). Amlodipine: an overview of its pharmacodynamic and pharmacokinetic properties. Clin Cardiol.

[13] Manrique YJ, Lee DJ, Islam F, Nissen LM, Cichero JA, Stokes JR (2014). Crushed tablets: does the administration of food vehicles and thickened fluids to aid medication swallowing alter drug release?. J Pharm Pharm Sci.

[14] Endrenyi L, Tothfalusi L (2013). Determination of bioequivalence for drugs with narrow therapeutic index: reduction of the regulatory burden. J Pharm Pharm Sci.

[15] Wishart DS, Knox C, Guo AC, Cheng D, Shrivastava S, Tzur D (2007). DrugBank: a knowledgebase for drugs, drug actions and drug targets. Nucleic Acids Res.

[16] Drug Delivery Foundation: BCS database. 2015. http://www.tsrlinc.net/search.cfm. Accessed 5 November 2018.

[17] Benet LZ, Broccatelli F, Oprea TI (2011). BDDCS applied to over 900 drugs. AAPS J.

[18] Carrier M-N, Garinot O, Vitzling C (2004). Stability and compatibility of tegaserod from crushed tablets mixed in beverages and foods. Am J Health Syst.

[19] Belard S, Isaacs W, Black F, Bateman L, Madolo L, Munro J (2015). Treatment of childhood tuberculosis: caregivers’ practices and perceptions in Cape Town. S Afr Paediatr Int Child Health.

[20] Best BM, Capparelli EV, Diep H, Rossi SS, Farrell MJ, Williams E, Lee G, van den Anker JN, Rakhmanina N (2011). Pharmacokinetics of lopinavir/ritonavir crushed *versus* whole tablets in children. J Acquir Immune Defic Syndr.

[21] Food and Drug Administration. Center for Drug Evaluation and Research, Use of liquids and/or soft foods as vehicles for drug administration: general considerations for selection and in vitro methods for product quality assessments - draft guidance for industry. 2018. https://www.fda.gov/downloads/Drugs/GuidanceComplianceRegulatoryInformation/Guidances/UCM614401.pdf. Accessed 6 October 2018.

[22] Fotaki N, Vertzoni M (2010). Biorelevant dissolution methods and their applications in in vitro-in vivo correlations for oral formulations. Open Drug Deliv J.

[23] Shah VP (2013). Progressive applications of dissolution, its impact, and implications in the pharmaceutical world. J Pharm Sci.

[24] Kostewicz ES, Abrahamsson B, Brewster M, Brouwers J, Butler J, Carlert S, Dickinson PA, Dressman J, Holm R, Klein S, Mann J, McAllister M, Minekus M, Muenster U, Müllertz A, Verwei M, Vertzoni M, Weitschies W, Augustijns P (2014). In vitro models for the prediction of in vivo performance of oral dosage forms. Eur J Pharm Sci.

[25] Guimarães M, Statelova M, Holm R, Reppas C, Symilllides M, Vertzoni M, Fotaki N (2019). Biopharmaceutical considerations in paediatrics with a view to the evaluation of orally administered drug products–a PEARRL review. J Pharm Pharmacol.

[26] BNF for Children (BNFC) 2016-2017: Royal Pharmaceutical Society of Great Britain, British Medical Association, Pharmaceutical Press; 2017.

[27] USP 30- NF 25. United States Pharmacopeia Convention. Rockville, USA; 2007.

[28] Fiolka T, Dressman J (2018). Development, current applications and future roles of biorelevant two-stage in vitro testing in drug development. J Pharm Pharmacol.

[29] Jensen J, Cornett C, Olsen CE, Tjørnelund J, Hansen SH (1992). Identification of major degradation products of 5-aminosalicylic acid formed in aqueous solutions and in pharmaceuticals. Int J Pharm.

[30] Smith GA, Rawls CM, Kunka RL (2004). An automated method for the determination of montelukast in human plasma using dual-column HPLC analysis and peak height summation of the parent compound and its photodegradation product. Pharm Res.

[31] Raju KN, Swamy TG, Rao AL (2011). Development and validation of RP-HPLC method for the determination of montelukast sodium in bulk and in pharmaceutical formulation. J Pharm Chem Biol Sci.

[32] Fadda H, Sousa T, Carlsson A, Abrahamsson B, Williams J, Kumar D (2010). Drug solubility in luminal fluids from different regions of the small and large intestine of humans. Mol Pharm.

[33] Wold S, Sjöström M, Eriksson L (2001). PLS-regression: a basic tool of chemometrics. Chemom Intell Lab Syst.

[34] Thibert R, Mach H, Clas S-D, Meisner DR, Vadas EB (1996). Characterization of the self-association properties of a leukotriene D4 receptor antagonist, MK-0476. Int J Pharm.

[35] Klein S, Shah VP (2008). A standardized mini paddle apparatus as an alternative to the standard paddle. AAPS PharmSciTech.

[36] Hopgood M, Reynolds G, Barker R (2018). Using computational fluid dynamics to compare shear rate and turbulence in the TIM-automated gastric compartment with USP apparatus II. J Pharm Sci.

[37] Baxter JL, Kukura J, Muzzio FJ (2005). Hydrodynamics-induced variability in the USP apparatus II dissolution test. Int J Pharm.

[38] Radwan A, Amidon GL, Langguth P (2012). Mechanistic investigation of food effect on disintegration and dissolution of BCS class III compound solid formulations: the importance of viscosity. Biopharm Drug Dispos.

